# Assessing Cerebellar Disorders with Wearable Inertial Sensor Data Using Time-Frequency and Autoregressive Hidden Markov Model Approaches

**DOI:** 10.3390/s22239454

**Published:** 2022-12-03

**Authors:** Karin C. Knudson, Anoopum S. Gupta

**Affiliations:** 1 Data Intensive Studies Center, Tufts University, Medford, MA 02155, USA; 2 Department of Neurology, Massachusetts General Hospital, Harvard Medical School, Boston, MA 02114, USA

**Keywords:** ataxia, wearable sensors, IMUs, Bayesian nonparametrics, time-frequency analysis, hidden Markov models, wavelets

## Abstract

Wearable sensor data is relatively easily collected and provides direct measurements of movement that can be used to develop useful behavioral biomarkers. Sensitive and specific behavioral biomarkers for neurodegenerative diseases are critical to supporting early detection, drug development efforts, and targeted treatments. In this paper, we use autoregressive hidden Markov models and a time-frequency approach to create meaningful quantitative descriptions of behavioral characteristics of cerebellar ataxias from wearable inertial sensor data gathered during movement. We create a flexible and descriptive set of features derived from accelerometer and gyroscope data collected from wearable sensors worn while participants perform clinical assessment tasks, and use these data to estimate disease status and severity. A short period of data collection (<5 min) yields enough information to effectively separate patients with ataxia from healthy controls with very high accuracy, to separate ataxia from other neurodegenerative diseases such as Parkinson’s disease, and to provide estimates of disease severity.

## 1. Introduction

Degenerative cerebellar ataxias are a heterogeneous group of disorders which include hereditary ataxias such as the spinocerebellar ataxias (SCAs), Friedreich’s ataxia (FRDA), and ataxia–telangectasia (AT), as well as acquired ataxias such as idiopathic late-onset cerebellar ataxia (ILOCA) and cerebellar-type multiple system atrophy (MSA). Cerebellar ataxias are relatively rare; AT affects 1–2.5 in 100,000, and the prevalence of the SCAs are estimated to range from 0.0 to 5.6 per 100,000 [1]. For comparison, Parkinson’s disease, another neurodegenerative disease with movement-related hallmarks, is estimated conservatively at 0.3% overall and >3% in those over 80 years old [2]. Symptoms of ataxia include loss of balance and coordination, slurred speech, loss of fine motor skills, and difficulty walking. Symptoms and rate of progression of ataxia vary by individual and by type of ataxia.

Sensitive, specific, and high resolution biomarkers for ataxia are necessary to support the development, deployment, and monitoring of (targeted) interventions as well as for supporting early diagnosis efforts. The heterogeneity and rareness of cerebellar ataxias add to both the challenge and the importance of developing such biomarkers.

Clinical scales are very important in the assessment of ataxia, and include SARA [3], ICARS [4], and the Brief Ataxia Rating Scale (BARS) [5]. Assessment via such scales requires a clinical visit, and progression from one discrete point to the next on the scale may happen slowly. Scores on these scales thus suffer from low resolution in two senses. First, the burden of a clinical visit means that there are generally months between repeat assessments. Second, potentially meaningful small degrees of progression in severity between visits may be missed due to scale coarseness and subjectivity.

There has been increasing interest in the use of wearable sensors such as inertial measurement units (IMUs) to record accelerometer and gyroscope data in assessing the progression of neurodegenerative diseases. A goal of such research is to develop characterizations of movement-related symptoms in individuals that can serve as biomarkers and complement existing clinical scales in describing and quantifying disease features [6]. Data from IMUs can provide direct information about movement. Such data can be used to provide fine-grained and nuanced descriptions of disease features and severity, allowing for a clearer picture of a disease’s natural history and response to treatment. Moreover, wearable sensor data from IMUs is gathered noninvasively and can be obtained in both a clinical setting and from patients at home [7]; the relative ease of collection underscores the potential of wearable sensor-based biomarkers to provide a higher temporal resolution than what is possible from in-person clinical scoring.

A hidden Markov model with hierarchical Dirichlet process priors and added state “stickiness” is a Bayesian nonparametric approach that can be used to segment sequential data (e.g., time series) and assign each segment to a latent state, the number of which need not be fixed beforehand [8]. Sticky HDP-HMMs extend and differ from standard hidden Markov models in several ways that make them especially relevant to the study of IMU data. The hierarchical Dirichlet process priors accommodate an undetermined number of latent states. Meanwhile, the “stickiness” increases the prior probability of self-transitions between latent states, meaning that a learned latent state is likely to persist through subsequent time points; dividing the time series each time the latent state transitions to a different state then provides a natural segmentation of the signal. Sticky HDP-HMMs have been further extended to include switching dynamics that govern the observed data points via autoregressive (AR) processes to form AR-HMMs in which each hidden state corresponds to different learned autoregressive dynamics [9]. That is, while sticky HDP-HMMs assume that the observations at each time point are conditionally independent given the hidden states, their AR-HMM extensions assume that each observation relates to the previous observation(s) via an autoregressive process and that the parameters governing the autoregressive process switch with the hidden state (e.g., contrast Figure 3c of [8] and Figure 1 of [9] for a graphical model view of the additional dependencies introduced with the AR-HMM). The resulting AR-HMMs could be well-suited to aid in the generation of rich and flexible representations of movement data, as they can be used to simultaneously learn to parse movements as well as the number of segment types and their dynamics. We evaluate their utility in this context in the current work.

Our contributions can be summarized as follows:

- We demonstrate that an autoregressive hidden Markov model (AR-HMM) that allows for nonparametric extension can be used to characterize and parse movement within clinical motor assessments recorded by IMUs. To our knowledge, this paper represents the first application of such models to human motion measured by IMUs.
- We present two approaches to developing meaningful and descriptive quantifications of movement tasks recorded by IMUs, with one set learned from data based on an autoregressive hidden Markov model and another based on a time-frequency approach.
- We apply these approaches to clinical data, demonstrating excellent classification accuracy and good severity score estimation accuracy for ataxia when the AR-HMM and time-frequency features are combined and used with classical random forest machine learning approaches.

### Related Work

As the technologies for wearable inertial sensors such as gyroscopes and accelerometers have become more advanced and widespread in recent years, research into their potential for characterizing and quantifying neurodegenerative diseases has increased. In particular, a growing volume of work has emerged around the potential of inertial wearable sensors in quantifying Parkinson’s disease; for reviews, see [10,11]. Inertial sensors have been used for gait analysis [12] and for the identification of tremor [13] and bradykinesia [14]. Inertial sensor data has shown potential in both identifying individuals with Parkinson’s [15] and quantifying its severity [16]. Studies have explored data gathering in clinical as well as in at-home settings [7,17,18].

The literature on inertial sensor data for quantifying ataxia (to which the present work contributes) is a notably smaller body of work, with the greatest area of focus thus far on measuring features of gait [19,20,21,22,23,24]. IMU measurements of rhythmic finger tapping with the finger and/or foot, as well as reaching tasks, have been used to predict the presence and severity of ataxia [25,26,27]. In the gait and finger tapping settings, orientation of the sensors relative to the surroundings stays relatively constant compared to the clinical tasks we consider in this work. Features used to train models to predict ataxia status and severity based on IMU data have included those based on multiscale entropy or fuzzy entropy [22,25,26], inter-movement intervals [25,28], and features calculated from the frequency domain [21,26]. In Parkinson’s disease, CNN-based approaches [14] and wavelet transform-derived features [29] have been used as well.

Initially applied to segment audio files for speaker diarization [8], sticky HDP-HMMs have been used in chunking human motion data from a motion capture system for imitation learning [30]. The extensions of such models that the current work makes use of, which incorporate learned dynamics governed by autoregressive processes [9], have been used to characterize the motion of mice (captured with video) as a combination of discrete movement modules [31] in ways shown to be informative, e.g., about drug identity, dose, and class, when diverse mouse behavior was generated via pharmacology [32].

## 2. Materials and Methods

### 2.1. Data Collection and Participants

Individuals with ataxia and Parkinsonism were recruited from Massachusetts General Hospital (MGH) Ataxia Center and Movement Disorders Unit. These individuals participated in the study either directly before or immediately after their neurology appointment at MGH. Participants were not instructed to adjust their usual medication dosing; thus, Parkinsonism participants taking levodopa were presumed to be in their “on” state. Children with ataxia–telangiectasia (AT) were recruited in collaboration with the Ataxia–Telangiectasia Children’s Project. Healthy control data were obtained from (1) family members of patients (e.g., asymptomatic partners or gene negative family members), (2) MGH staff, and (3) “Rally with Mass General Brigham”, which is an online resource that healthy volunteers can use to find research studies at MGH. For analysis, we used only subjects from whom we had recordings for for all three tasks, namely, finger-nose-finger, alternating hand movements, and heel-shin.

Participants wore six APDM sensors: one sensor on each wrist and ankle, a sensor in the pocket, and a lumbar sensor. The wearable sensors captured data at a sampling frequency of 128 Hz along three axes from an accelerometer, gyroscope, and magnetometer for a total of nine channels per sensor as the participants performed a variety of clinical tasks.

The analysis in this work used accelerometer and gyroscope data during three tasks that are commonly performed as part of a neurological examination. The finger-nose-finger task involves iteratively reaching to touch a target on a tablet computer screen followed by the participant’s nose. Finger-nose-finger was performed for 40 s with each hand. The “fast alternating hand movements” task involves rapid pronation and supination of the arm with the arm extended out in front of the participant and with a slight bend at the elbow and slight extension at the wrist. This task was performed for 10 s with each hand. The heel-shin task involves sliding the heel of one foot along the shin of the other foot repetitively from the knee to the ankle. This task was performed for 20 s with each leg. We analyzed the signal from the wrist sensors for the finger-nose-finger and alternating hand movements tasks and from the ankle sensors for the heel-shin task; the lumbar and pocket sensor data were omitted as less relevant to these tasks. These tasks were selected from among those performed because they together represent upper and lower extremity function and because the motions associated with them are rhythmic and repetitive, and thus our choices of frequency-based characteristics for the time-frequency portion of the analysis were naturally applicable.

### 2.2. Preprocessing

For each individual’s performance of a task, principal component analysis was used separately on the three-dimensional accelerometer and gyroscope signals from each sensor and the loadings for the first principal component were retained. This operation reduced the dimensionality of the signal threefold at each time point and removed the dependence of the signal on sensor orientation. Signals were divided temporally into equal ‘rest’ and ‘task’ portions based on the total magnitudes of velocity in the signal; for example, a sensor on the right lower extremity during the heel-shin task included the ‘task’ portion when the task was performed with the right leg and the ‘rest’ portion when the task was being performed with the left leg. The reason for this division between ‘task’ and ‘rest’ was according to the total magnitudes of velocity in the signal for each individual and task, and was intended to to account for occasional inconsistencies when a subject started their motion on the opposite side from what was instructed.

For the AR-HMM based features, we additionally included a wavelet denoising step with a Symlets-4 wavelet and a threshold of 0.04 on the vector of loadings of the first principal component [33]; to speed computation in the statistical learning of the AR-HMM, we downsampled each time series by a factor of ten.

### 2.3. Time Frequency Features

**Figure 1:**
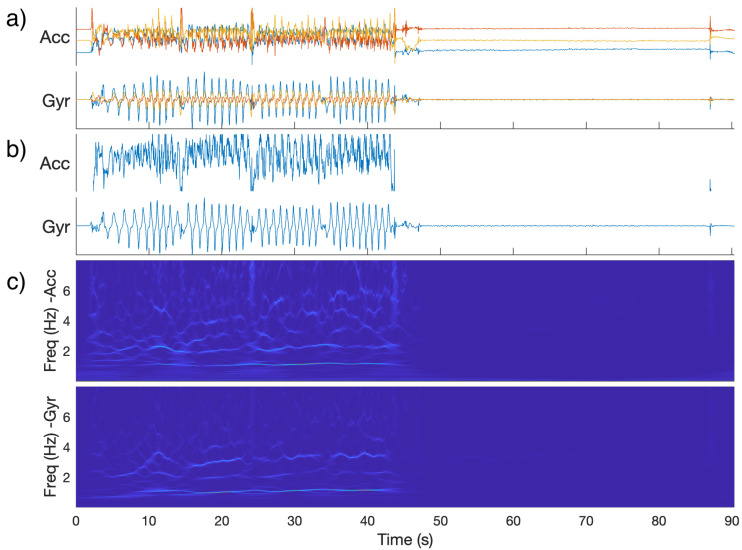
(**a**): Raw IMU signals for a control subject performing the finger-nose-finger task with their right hand (one color per component). (**b**): Signals projected along first principal component. (**c**): Magnitude of synchrosqueezed transform (SST) of projected signals. Note: During the right half of the graphs, the measured hand was at rest. On the left-hand side, a noticeable feature is the group of horizontal lines around 1 Hz, which is the approximate frequency with which the subject completed the repeated motion.

For the time-frequency based features, a wavelet synchrosqueezed transform [34] with an analytic bump wavelet was applied to a one-dimensional data vector for each combination of session and task, sensor position (right or left relevant extremity), and modality (accelerometer or gyroscope) to yield a time-frequency distribution of power for each signal (Figure 1) [35]. Compared to a wavelet transform, the wavelet synchrosqueezed transform includes a “reallocation” along the frequency axis that tends to sharpen the result in the frequency direction. In the context of rhythmic tasks and neurodegenerative diseases that can affect the rhythmicity and speed of actions, such sharpness in the frequency direction is desirable. Frequencies above 15 Hz were omitted from the analysis.

The following 16 features were calculated for each combination of task (N = 3), sensor position (N = 2, left and right side), and sensor modality (N = 2, accelerometer and gyroscope), for a total of 192 features. Note that several features are calculated using frequency thresholds: above or below 2 Hz for finger-nose-finger and heel-shin, and 3 Hz for alternating hand movements. These cutoffs were chosen because the frequencies of the tasks themselves (e.g., the rate of reaching in the finger-nose-finger task and rotating in the lightbulb task) were deemed to be lower than 2 or 3 Hz, respectively, while frequencies of a tremor would generally fall above this cutoff.

- Total power (rest and task): Power summed across all time bins and all frequencies.
- Ratio of task to rest power: Total power during task divided by total power during rest.
- Low frequency power (rest and task): Power summed across all time bins and all frequencies below the cutoff (2 or 3 Hz, depending on task).
- High frequency power (rest and task): Power summed across all time bins and all frequencies above cutoff.
- Ratio of low to high frequency power (rest and task): Low frequency power divided by high frequency power.
- Center frequency (rest and task): Weighted sum of frequency (with weights the total power for that frequency summed over time), divided by summed power over all frequencies.
- Spread of frequency (rest and task): Squared difference of frequency from center frequency, multiplied total power for that frequency and then summed over all frequencies.
- Center frequency of low frequency (task): As above, but calculated only for frequencies below cutoff.
- Center frequency of high frequency (task): As above, but calculated only for frequencies above cutoff.
- Cosine similarity of adjacent time bins (task): Mean cosine similarity between the vectors of powers for each frequency for adjacent time bins.

These features were designed before the downstream analysis was performed and were chosen to capture known features of ataxia, such as changes in the rhythmicity of movement.

### 2.4. Autoregressive Hidden Markov Model

To construct a second complementary set of features, we turned to a different paradigm.

**Figure 2:**
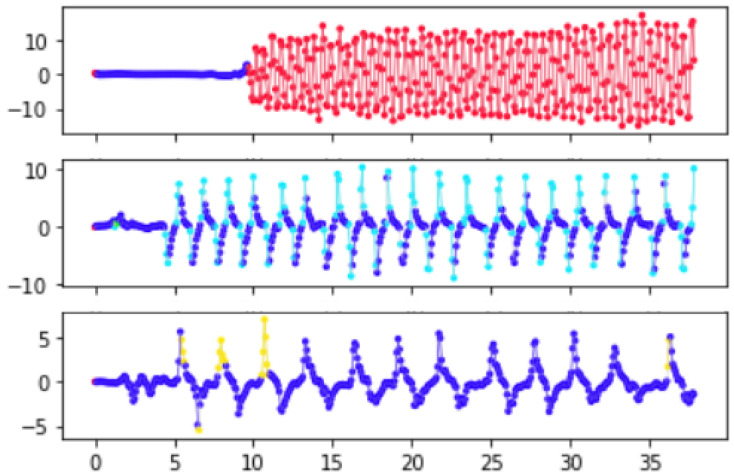
An example time series in which each time point in a preprocessed gyroscope signal has been assigned a learned latent state (state represented by color). (**Top**): alternating hand movements task (subject with mild ataxia). (**Middle**): finger-nose-finger (control subject). (**Bottom**): finger-nose-finger (subject with ataxia).

In the AR-HMM model each discrete time point is assumed to have an unobserved underlying state, with the state evolving according to a Markov chain governed by unobserved probabilities of transitions between states at each point. Given the discrete state associated with a time point, the observed data (accelerometric and gyroscopic) are assumed to proceed via an autoregressive process for which the parameters are characteristic of that state. Thus, broadly speaking, when we perform inference to learn the sequence of states for motion data of a particular individual and task, each learned state is a label related to the characteristics of the motion in the preceding time bins (Figure 2). Here, the Markov model that governs transitions between states includes a stickiness parameter [8], which boosts the number of self-transitions to ensurer that states tend to persist over multiple consecutive time points, making model more suitable for segmenting the signal. Importantly, the states and their associated dynamics are learned from the data and state learning can proceed non-parametrically, meaning that the number of states needed to effectively describe the data can be learned from the data. Thus, applying this model to motion data can provide us with a picture of the motion as a sequence of segments, with each segment corresponding to a period of time in which the latent state is constant and the motion evolves according to the particular dynamics associated with that state.

Concretely, the model for the states $x_{t}$ and the observed multidimensional (here, two-dimensional after pre-processing) IMU data vectors ${\vec{y}}_{t}$ (with both *x* and $\vec{y}$ indexed by time) is provided by

$$p\left(x_{t}|x_{t-1},\pi \right)=\pi_{x_{t},x_{t-1}}$$

$${\vec{y}}_{t}|A^{\left(x_{t}\right)},\Sigma^{\left(x_{t}\right)},Y_{t-1:t-n},x_{t}∼$$

$$\mathrm{MVN}(A^{\left(x_{t}\right)}Y_{t-1:t-n},\Sigma^{\left(x_{t}\right)})$$

Here, π is the L×L transition matrix, where *L* is the number of discrete states and $x_{t}\in \left\{1,\ldots ,L\right\}$; the parameters $A^{\left(x_{t}\right)},\Sigma^{\left(x_{t}\right)}$ are derived from a learned set of *L* pairs

$$\left(A^{\left(1\right)},\Sigma^{\left(1\right)}\right),\ldots \left(A^{\left(L\right)},\Sigma^{\left(L\right)}\right)$$

that govern the dynamics; and MVN denotes the multivariate normal distribution. By $Y_{t-1:t-n}$, we denote the observed data vectors from the *n* time points preceding time *t*. For the present application, we set the number of time lags in the model to n=5, meaning that the next time point evolves in accordance with the previous 0.4 s of data. We explored n=4 and n=6 lags, which provided results that appear qualitatively similar. Across a broader range, however, we expect the choice of the number of lags to have a strong effect on the model as well as on the choice of other parameters; for example, adding more lags allows additional complexity in the dynamics, suggesting that a corresponding increase in the number of latent states might be needed in order to capture this complexity.

We are following procedures common in other applied uses of HDP-HMMs by truncating to a finite number of states *L* and using Dirichlet distribution priors [8,9,31]. Note that as L→∞ the finite hierarchical model converges in distribution to a hierarchical Dirichlet process prior [36].

Let κ be a stickiness parameter that is chosen empirically (a larger κ encourages runs of consecutive time points in the same state), and let ${\vec{\delta }}_{i}$ be the unit vector such that $\delta_{i,j}=1$ when i=j and is 0 otherwise. We assume the following prior distributions for π,A,Σ:

$$A,\Sigma ∼\mathrm{MNIW}(\nu_{0},S_{0},M_{0},K_{0})$$

$$\vec{\beta }∼\mathrm{Dirichlet}\left(\gamma /L,\ldots \gamma /L\right)$$

$${\vec{\pi }}_{i\cdot }∼\mathrm{Dirichlet}\left(\alpha \vec{\beta }+\kappa {\vec{\delta }}_{i}\right).$$

where MNIW denotes the matrix normal inverse Wishart distribution. For better intuition into the role of $\alpha ,\kappa ,\vec{\beta }$, note that the $j^{th}$ component of $\alpha \vec{\beta }+\kappa {\vec{\delta }}_{i}$ provides the prior pseudocounts of transitions from state *i* to state *j*. Thus, β controls the prior relative frequencies of transitions to each state before the $\kappa {\vec{\delta }}_{i}$ is added in order to increase the prior probability of self-transitions.

We set the hyperparameters $\nu_{0}$, $S_{0}$, $M_{0}$, $K_{0}$, α, γ, and κ as follows, although these hyperparameters could be given their own prior distributions if desired. We let α=γ=κ=20 based on prior predictive sampling providing runs of states (interpreted as segment length) that were centered around 100–200 ms in length (median run length of 156 ms on a 30 s simulated interval). We set L=5, because with these hyperparameter values and larger number of states (6 or 8) over 98% of states in the training data were assigned to one of the five most common states. In adapting to other contexts, these hyperparameters could be changed to encourage more states, or alternatively the model could be extended to allow for an indeterminate number of states. In our current setting, we were content with a small number of states for which the associated dynamics could be more easily inspected. We set the hyperparameters for the autoregressive parameters to $\nu_{0}=5,\;S_{0}=0.01I,M_{0}=0.251_{2\times 2n}$, where I denotes the identity matrix and $1_{2\times 2n}$ denotes the n×2n matrix of all ones, and we let $K_{0}$ be a diagonal matrix with entries linearly spaced between 5 and 100.

It is important to highlight several assumptions implicit in applying this model. First, we note that the terms that control the dynamics A,Σ depend on the time point only through the value of latent state $x_{t}$, not through *t* itself. Thus, we can identify a set of autoregressive parameters $\left(A^{\left(1\right)},\Sigma^{\left(1\right)}\right),\ldots \left(A^{\left(L\right)},\Sigma^{\left(L\right)}\right)$ that among them describe the dynamics throughout the time series and (in the present application) across sensors and task motions. Second, we assume that there is not an additional trend or bias term in the AR process. More precisely, we note that one could use the same inference process while including an additional state-dependent term in the dynamics governing the observations, such that $Y_{t-1:t-n}$ would be centered around $A^{\left(x_{t}\right)}Y_{t-1:t-n}+b^{(}x_{t})$ instead of simply $A^{\left(x_{t}\right)}Y_{t-1:t-n}$, as is the case here.

**Figure 3:**
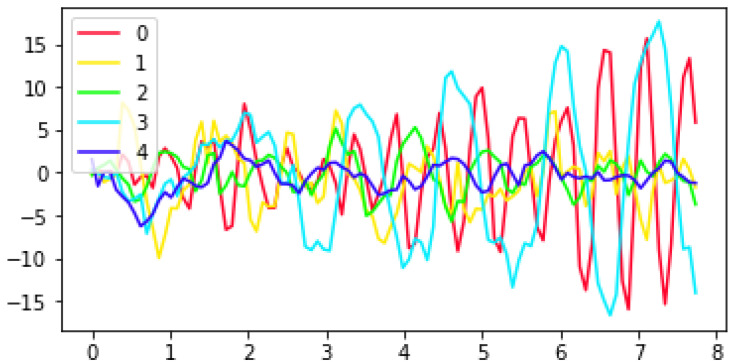
Sample trajectories evolving according to the estimated AR parameters $\left({\vec{A}}^{\left(0\right)},{\vec{\Sigma }}^{\left(0\right)}\right),\ldots \left({\vec{A}}^{\left(4\right)},{\vec{\Sigma }}^{\left(4\right)}\right)$ for each of the five states. Visual inspection of such trajectories are a simple tool to help add insight about the types of movement corresponding to each state. For instance, the sample trajectory from state 0 exhibits the kind of very fast oscillations that were more common in the alternating hand movements task in control or mildly ataxic subjects than in subjects with higher BARS scores.

Our inference for the posterior distributions proceeded via Gibbs sampling (see [9,31] for computation of the full conditionals required for Gibbs sampling). However, in order to reduce computation time the parameters A,Σ that controlled the autoregressive process were first learned (via Gibbs sampling) from a subset of the data that was chosen to represent the range of common movement types. This subset of data included a recording from each of the three tasks for four subjects (a control and three ataxic subjects) with BARS scores ranging from 1 to 20. We sampled to obtain 2000 draws and used a burn-in of 100 samples. Mode switching was a concern, as there are symmetries in the labeling of the states and the posterior means of the autoregressive parameters feed into the next step of sampling. Visual inspection of the sampled β parameters showed an obvious mode switch around 1000 samples in; thus, we truncated to the first 1000 samples (with a burn-in of 100 samples) when computing the posterior means of the autoregressive parameters. See Figure 3 for sample trajectories evolving according to the the learned dynamics associated with each state as measured by their posterior means.

We then proceeded with the posterior means of $\left(A^{\left(1\right)},\Sigma^{\left(1\right)}\right),\ldots ,\left(A^{\left(L\right)}\right),\Sigma^{\left(L\right)})$ as fixed autoregressive parameters (using the posterior means for β and π from the first stage of sampling as initial values in the Gibbs sampler) and learned the state sequences $x_{t}$, transition probabilities, $\vec{\pi }$, and parameters $\vec{\beta }$ for each individual, task, and sensor. In this second stage of Gibbs sampling, in which the state-related parameters are learned, we drew 200 samples, with a burn-in of 50 samples.

The following features were calculated from each participant’s sequence of state sample modes for each behavioral task:

- The frequencies of each state in the data
- Each state’s estimated self-transition probability
- The mean of the the length of runs (consecutive appearances) of each state
- The standard deviation of the length of runs (consecutive appearances) of each state
- The estimated entropy rate of the state sequence as calculated from the posterior mean of the Markov transition probabilities.

We used both the state samples and their modes to calculate two more features aimed at quantifying the degree to which drawn samples were concentrated around the mode:

- For each state, the proportion of state samples drawn that were equal to that mode was averaged over all time points for which that state was the mode
- The proportion of all state samples that were equal to their corresponding mode state in time.

We thus calculated five state-specific features (for each of L = 5 states) and two other features, for 27 features from each of N = 3 tasks and N = 2 sensors (left and right), yielding a total of 162 AR-HMM derived features.

**Figure 4:**
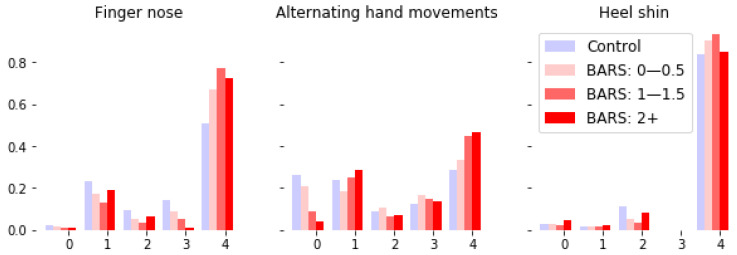
Frequency of occurrences of the latent states (numbered 0–4) present in sessions corresponding to different tasks and BARS subscores (BARS right arm subscore for finger-nose-finger and alternating hand movements, BARS right leg subscore for heel-shin). The dynamics associated with a state are common across all tasks. Note the variation in frequency of state based on task and severity of ataxia score. For instance, within the alternating hand movements task there seems to be a systematic trade off between the frequency of occurrence of states 0 and 4 as the BARS arm score increases.

Many other derived features are possible; we selected the current set in order to form a baseline and demonstrate the broad applicability of the AR-HMM-based coding of states to disease status and severity. As a visual check of the relevance of the coded states, we visualized sample trajectories for each state (Figure 3). Note that the learned frequency of each state vary by task and diagnosis (Figure 4).

### 2.5. Classification

Classification of disease status, such as ataxia vs. control or ataxia vs. Parkinsonism, was performed using a balanced random forest with 200 trees. Balanced random forest modifies a random forest with undersampling to compensate for the imbalanced classes; here, the imbalance was that there were more recordings from ataxic subjects than from control subjects [37,38].

### 2.6. Score Prediction

BARS scores were estimated by training a random forest model with 200 trees, a maximum tree depth of 10, and a mean absolute error (MAE) criterion [39]. Predictions were performed for BARS total score (obtained for 133 out of 183 sessions) and two motor subscores: the BARS right arm score (obtained for 155 out of 183 sessions) and an arm-and-leg subscore consisting of the sum of the BARS arm and leg score (obtained for 142 out of 183 sessions, included due to a of a lack of gait scoring for certain subjects). Total BARS scores (which sum oculomotor and speech subscores with clinical scores related to arms, legs, and gait) range from 0 to 30, with higher numbers indicating greater severity. The BARS right arm subscore ranges from 0 to 4, while the sum of the arm and leg subscores ranges from 0 to 16.

### 2.7. Cross-Validation

All results were computed and reported based on a leave-one-subject-out cross-validation setup, performing the random forest training while all session data from one subject was held out, using the selected model to predict the disease status or severity score for the session(s) for that one subject, and then aggregating measures of the correctness of the predictions across all the predictions for held-out subjects in order to report the relevant accuracy metrics.

### 2.8. Code Availability

The code for our methods is available at: https://github.com/karink520/sensor_util_ataxia_public (accessed on 30 September 2022).

## 3. Results

Data were collected from 212 participants and 264 sessions (with repeat sessions for a number of subjects, typically recorded months apart). Of these participants, 195 individuals participating in 242 sessions provided recorded data from all three motor tasks. These included 109 individuals with ataxia, 52 individuals with Parkinsonism, and 34 controls; see Table 1 for clinical and demographic information.

### 3.1. Classification Results

**Figure 5:**
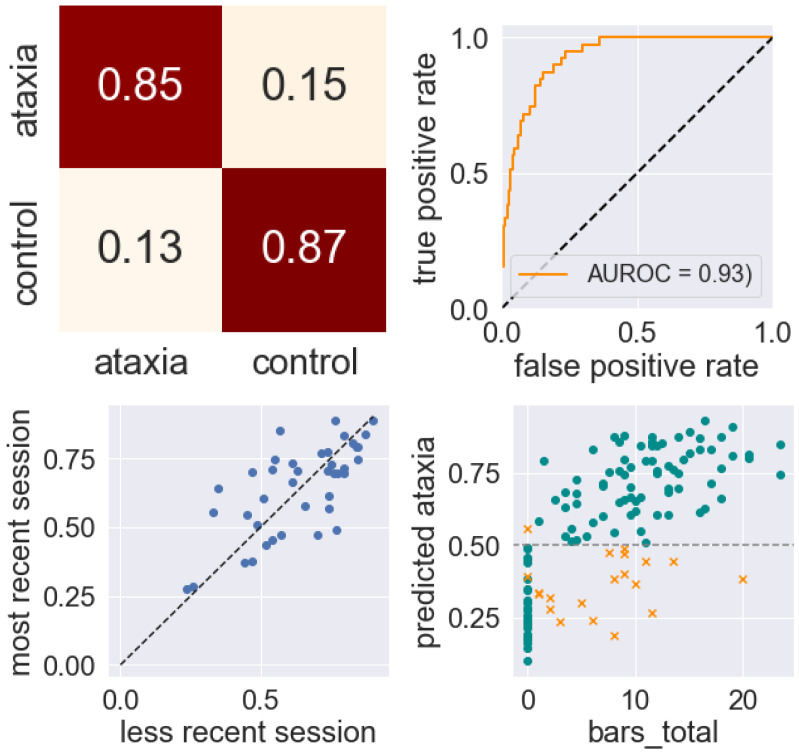
Classification performance on distinguishing ataxic subjects from control subjects. From left to right and top to bottom: normalized confusion matrix (rows show true classification, columns show prediction); ROC curve; test–retest correlation of classification scores, with scores closer to 1 representing the prediction of ataxia and scores closer to 0 the prediction of control; Total BARS score vs. predicted probability of ataxia (>0.5 corresponds to a prediction of ataxia, x = incorrect prediction).

Classification results were highly accurate, with classification of ataxia against controls achieving an area under the ROC curve (AUROC) of 0.93, a sensitivity of 0.85, and a specificity of 0.87. (Figure 5 and Table 2). The test–retest correlation of classification scores calculated from the 35 sessions of subjects who had repeat visits was 0.72.

The relative importance of the time-frequency and AR-HMM based features was evaluated. When the two feature sets were used together, the most highly informative features were a mix of features from both sets. Out of the top 20 features by importance in the classifier (ataxia vs. control), eleven features come from the time-frequency approach and nine come from the AR-HMM approach. Thirteen of these top 20 features came from the finger-nose-finger task, seven from the alternating hand movements task, and none from the heel-shin task. When classification was performed using only features from the time-frequency approach, the AUROC for the ataxia vs. control classification task was 0.93 (sensitivity: 0.86, specificity: 0.90). When classification was performed using only features derived from the AR-HMM approach, the AUROC for the ataxia vs. control classification task was 0.89 (sensitivity: 0.81, specificity: 0.80).

Because one potential use for wearable sensor-based measurement of disease is early detection, we considered the results of training a classifier to detect ataxia when the set of subjects is limited to mild cases (here, we take mild to mean having a total BARS score of less than 10). For this task, the AUROC score was 0.87 (sensitivity: 0.76, specificity: 0.82). In addition, we separately considered the classification tasks for adult ataxia vs. control and pediatric ataxia vs. control. Here, we define subjects under age 18 as pediatric. Performance of the classifier was better when limited to pediatric subjects, most of whom had ataxia–telangiectasia (AT) and more severe ataxia.

**Figure 6:**
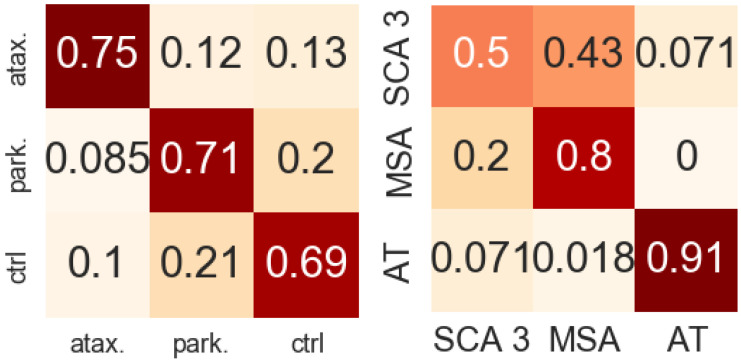
Left: normalized confusion matrix for classification performance distinguishing ataxia, Parkinsonism, and control. Right: classification of three specific ataxia diagnoses. Rows are true classification, columns are predicted classification.

Classification of ataxia vs. Parkinsonism was highly accurate (AUROC 0.93: sensitivity 0.88: specificity: 0.83), suggesting that in addition to simply demonstrating overall motor impairment, the features described above can describe the type of impairment. In the ternary classification ataxia vs. parkinsonism vs. control subjects, we observed an accuracy of rate of 0.70 (Figure 6).

We assessed the applicability our feature set to the task of distinguishing between different types of ataxia. In the task of classifying ataxia–telangiectasia (AT), multiple system atrophy (MSA), and spinocerebellar ataxia type 3 (SCA 3, the most well represented of the SCAs in our data), we obtained an accuracy of 0.80. (Figure 6).

### 3.2. Score Prediction Results

Our model predicts BARS total scores and subscores as described in Section 2.6 based on training on the combined set of all control subjects (who had BARS scores of 0) and all subjects with ataxia.

**Figure 7:**
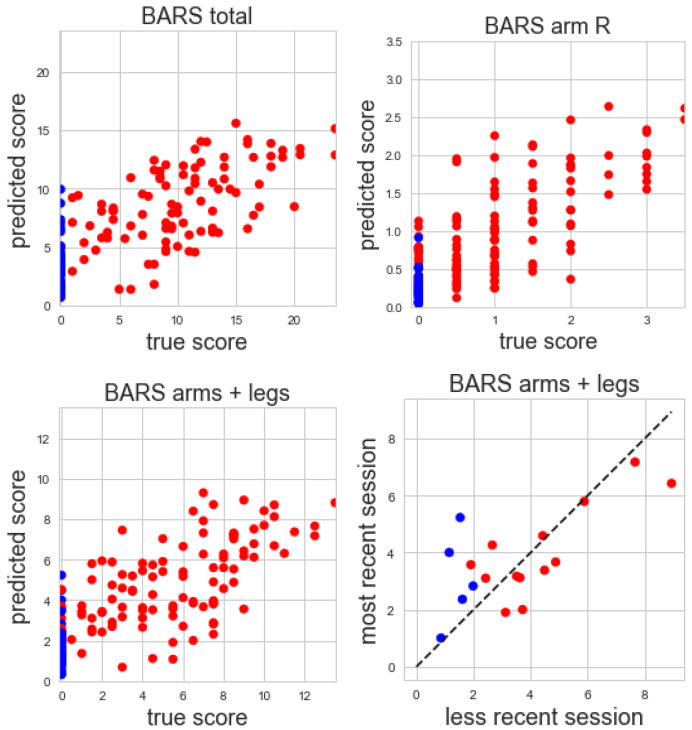
From Top left: true vs. predicted total BARS scores; top right: true vs. predicted BARS right arm subscore; bottom left: true vs. predicted BARS arm and leg subscores; bottom right: test–retest reliability of BARS arm and leg subscores for subjects with multiple visits (red = ataxia, blue = control).

The correlation of predicted and true total BARS score is 0.74 ($R^{2}=0.53$), with a mean absolute error (MAE) of 3.76 points and a test–retest correlation 0.70 from 16 subjects with multiple visits. The correlation of predicted and true BARS motor arm and leg subscore is 0.75 ($R^{2}=0.54$, MAE = 2.14, test–retest correlation 0.70 from 16 subjects with multiple visits). The correlation of predicted and true right arm BARS score is 0.77 ($R^{2}=0.59$, MAE = 0.47, test–retest correlation 0.96 from 20 subjects with multiple visits) (Figure 7).

## 4. Discussion

Our results demonstrate the relevance of the two approaches to representing movements measured by IMUs in the context of describing neurodegenerative diseases, particularly cerebellar ataxias. Respectively, the two approaches begin by performing a synchrosqueezed wavelet transform and calculating features from the time-frequency domain, then training an AR-HMM to learn segmentation of the time series data. Both approaches produce features that are relevant to predicting an individual’s diagnosis and disease severity. Moreover, we discuss the distinct value of the two approaches as they differ in their applicability and generalizability. In particular, we emphasize that many of the the time-frequency based features are most applicable to rhythmic tasks based on repeated movement, such as the ones considered here, while we expect the more task-agnostic AR-HMM-based segmentation of motion to generalize to broader classes of motion.

We find that the features derived from each approach are highly informative about an individual’s diagnosis, even in the context of short data recordings (under 5 min per participant). In addition to allowing for high rates of success in classifying ataxic subjects against healthy controls, models trained on these features were able to perform several more subtle tasks such as classifying mild ataxia and healthy controls, classifying ataxia and Parkinsonism, and distinguishing several types of ataxia. Additionally, we found that regression models trained on AR-HMM and time-frequency features had good performance in estimating disease severity as measured by neurologist-administered clinical scales.

We emphasize that the success of these models in distinguishing ataxia from Parkinsonism and in distinguishing different types of ataxia is noteworthy; it suggests that the features are able to quantify aspects of subjects’ movements beyond merely detecting broad impairment.

The short length of the recordings used with the models is of considerable clinical relevance, as it can translate to a reduced burden on subjects who are asked to perform clinical tasks.

The performance of the classifier on these features is comparable with the best performance we have seen in the literature. In related work applying a time-frequency approach to rhythmic tapping, an AUC of 0.90 was reported for classifying ataxia vs. controls [26]. Research conducted simultaneously with the present work that involved decomposing reaching movements reported an AUC of 0.96 and 0.89 for classifying ataxia vs. control and ataxia vs. Parkinsonism, respectively [27]. We did not encounter movement data-based classifications of different types of ataxia (e.g., MSA vs. SCA 3) in the literature that are comparable to ours. We compared our severity estimation results to another very recent work [24] which used gait analysis and reported a $R^{2}$ value of 0.72 for total BARS prediction and 0.45 for upper extremity BARS score prediction (that is, a higher $R^{2}$ value for the total score than we found using our approach, and a lower score for the upper extremity, as might be expected for a gait-based analysis).

We used IMU measurements from three different clinical tasks. The inclusion of these three tasks and both upper and lower extremity sensors was important to our aim of investigating flexible and extensible feature-generating approaches that are not tailored to one precise task and are instead able to generalize across a range of movement types and sensor locations. For instance, we showed how AR-HMM was able to decompose movement into segments of latent movement dynamics that can be shared across sensor locations and tasks, and the same time-frequency based process for deriving features was applied in these different settings as well. Creating measures such as these that are able to capture information from diverse behavioral domains is important in characterizing ataxia, because an individual may not be uniformly affected across motor domains. The ability to assess the overall behavioral phenotype (rather than movement in just one limb, for example) is important in clinical trial settings, where overall improvement is the goal.

Although including several tasks was important to the aims of this investigation for the reasons mentioned above, we note that there may be ways to further reduce recording times for use in settings where brevity is of the highest importance. In particular, our results suggest that the alternating hand movements and finger-nose-finger clinical tasks were more informative than the heel-shin task for classification and severity prediction when using the chosen feature sets. It is possible that the assumed timescale of submovements was used to set model hyperparameters was not as appropriate for the (slower to complete) heel-shin task as for the other two tasks. Additional work with multiple clinical tasks and other kinds of movement is important in determining which types of movements and tasks can be used to most efficiently gather information about pertinent behavioral aspects of an individual’s neurodegenerative disease.

The time-frequency features derived from the synchrosqueezed transform were used to train effective classifiers on their own and in combination with the AR-HMM-based features. Our time-frequency approach is closer to existing techniques for gaining insight into neurodegenerative diseases via wearable sensor measurements. For example, the FFT has been used to extract gait cadence for assessing gait ataxia [21]. To quantify aspects of movement speed and rhythm ataxia, time-frequency features such as the coefficient of variation of inter-movement intervals or resonant frequencies and their amplitudes have been used [26]. Most comparably to the current work, a wavelet-based approach (used to calculate features of wavelet energy and entropy) has been applied to IMU data for assessing Parkinson’s disease [29]. The use of the synchrosqueezed transform as a starting point and the particular choice of the derived feature set used here is novel, and chosen with an eye towards simplicity and interpretability; we hope its use can add to the body of understanding regarding the features relevant to this domain.

Using a movement segmentation approach learned with an AR-HMM is novel in the setting of human IMU data and neurodegenerative diseases, and our results point to its applicability in this context. While the time-frequency-derived features appeared to provide slightly more accurate classifications when used on their own than the AR-HMM-based features, the highly adaptable nature of the AR-HMM approach makes it more likely to find application in a wider range of settings. A major advantage of AR-HMMs is their extensibility and flexibility. In the current work, we limited ourselves to a small number of states for the sake of interpretability (e.g., to ensure that we could visually inspect the dynamics associated with each state) and computational speed. Our preliminary explorations suggested that even with only these three relatively simple movement tasks the small number of states seemed sufficient. However, AR-HMMs can be extended nonparametrically to learn an appropriate number of states, and thus logically extended to encompass more kinds of movement (including movements that might not be well described by time-frequency-derived features). We emphasize that the AR-HMM features are task-agnostic, facilitating the future incorporation of additional behavioral tasks and even free-living behavior. Thus, as large-scale wearable sensor datasets emerge through current and future natural history studies, we believe that this class of models, being trained to identify and characterize more latent dynamical states, may facilitate improved early detection of neurodegenerative diseases, improved measures of disease progression, and the ability to characterize heterogeneity of dynamical states within a population, ultimately leading to identification of subgroups. This requires larger-scale datasets; however, this work demonstrates a potential path towards achieving that longer-term aim.

We note several additional limitations of our computational approaches. While the general contours of a time-frequency approach are broadly applicable for characterizing disease using movement data, several of the specific features we extracted from the time-frequency domain were designed to particularly exploit aspects of motions that are rhythmic and repeated, and may be less powerful in settings where movement lacks this structure. The AR-HMM features do not share this requirement, and are therefore more flexible in this sense. However, as mentioned above, in limiting our domain of investigation with the AR-HMM to a smaller number of hidden states, we limited the types of latent movement dynamics that could be learned by our AR-HMM. A broader exploration of different hyperparameter regimes (including the possibility of an additional bias term for observation dynamics, as mentioned in the methods section above) would be helpful in applying this approach to a more varied set of movements. Relatedly, our two-step procedure of training the full model on a subset of the data, then fixing the learned dynamics and learning the states for the rest of the data might limit the number of types of movement dynamics that can be learned, as the model could potentially miss a class of dynamical states that did not appear in the smaller subset of data.

Future applications of AR-HMMs in the context of IMU data and neurodegenerative diseases could involve deeper investigation into the choices of hyperparameters or hyperpriors that are appropriate in the setting of IMU data from a range of clinical tasks, as well as into capturing more varieties of movement within broader sets of tasks or potentially free-living behavior.

## 5. Conclusions

Our results demonstrate the relevance of the presently described AR-HMM and time-frequency-based approaches to representing movements measured by IMUs in the context of describing neurodegenerative diseases, particularly cerebellar ataxias.

The two approaches to feature construction presented here were chosen to be flexible, interpretable, and extensible. The features allowed for highly accurate categorization of diagnosis from less than five minutes of data when combined with a standard classical random forest machine learning approach.

Both the synchrosqueezed transform and the AR-HMM can serve as starting points for a rich set of features beyond what we consider here. Future work could consider our two approaches as pre-processing steps and explore the most relevant derived features in different settings and for different purposes. The current work points to the potential of these two classes of features derived from wearable sensor data to support important applications, for instance, as biomarkers to aid in understanding an individual’s disease and its progression and to support targeted interventions, effective clinical trials, and early diagnosis.

## Figures and Tables

**Table 1:** Demographic characteristics of subjects. Specific ataxia diagnoses are shown in separate lines for each diagnosis represented by more than one subject. Note that mean severity score for Parkinsonism is reported on the UPDRS [40], while ataxia severity scores are on the BARS scale. Ataxia (other) includes: spinocerebellar ataxia type 8, type 14, and type 15; cerebellar-dominant progressive supranuclear palsy; hereditary spastic paraplegia; autoimmune-related ataxia with undefined cause; Behcet’s Disease; autosomal recessive cerebellar ataxia type 1 and type 3; cerebellar hypoplasia; sensory ataxia; Fragile X-Associated Tremor/Ataxia Syndrome; Gordon Holmes’ Syndrome; stroke-related ataxia; sporadic adult-onset ataxia; sporadic adult-onset ataxia with neuropathy; autosomal dominant cerebellar ataxia with unidentified genetic cause.

	n	Age	Women	Men	Severity Score
	Subjects, Sessions	Mean (SD)			Mean (SD)
Total	195, 242	46.9 (24.1)	78	117	n/a
Ataxia (all)	109, 144	43.1 (23.8)	49	60	10.3 (5.3)
AT	34, 56	12.4 (6.1)	13	21	11.3 (5.7)
Episodic Ataxia	2, 2	50.0 (28.0)	0	2	1.2 (0.2)
FA	2, 2	55.5 (5.5)	1	1	16.2 (2.8)
MSA	5, 6	60.4 (5.2)	2	3	12.2 (3.8)
SCA 1	4, 8	49.0 (14.1)	3	1	8.1 (2.0)
SCA 2	2, 2	61.0 (6.0)	1	1	12.0 (1.5)
SCA 3	11, 14	51.0 (10.6)	9	2	9.5 (3.3)
SCA 6	8, 9	69.0 (6.0)	3	5	12.8 (6.1)
SPG 7	2, 2	58.0 (2.0)	0	2	9.0 (1.0)
Transient Ataxia	2, 2	72.5 (2.5)	1	1	1.0 (0.0)
Ataxia (other)	36, 40	55.8 (14.0)	16	20	10.6 (5.7)
Parkisonism	52, 59	67.6 (7.9)	14	38	16.5 (9.7)
Control	34, 39	27.5 (18.4)	15	19	n/a

**Table 2:** Metrics for binary classification of general diagnosis.

	AUROC	Sensitivity	Specificity	Test-Retest Correlation (# Sessions)
Ataxia/control	0.93	0.85	0.87	0.70 (35)
Ataxia/control (AR-HMM only)	0.89	0.81	0.80	0.51 (35)
Ataxia/control (time-freq only)	0.93	0.86	0.90	0.74 (35)
Mild ataxia/control	0.87	0.76	0.82	0.62 (12)
Mild ataxia/control (AR-HMM only)	0.80	0.78	0.78	0.61 (12)
Mild ataxia/control (time-freq only)	0.87	0.78	0.79	0.52 (12)
Pediatric ataxia/control	0.95	0.90	0.93	0.84 (20)
Adult ataxia/control	0.90	0.80	0.80	0.46 (15)
Ataxia/parkinsonism	0.93	0.88	0.83	0.94 (35)

## Data Availability

The data presented in this study are available on request from the corresponding author. The data are not publicly available due to privacy considerations in this rare disease population.

## Abbreviations

The following abbreviations are used in this manuscript:

APDM	APDM Wearable Technologies
AR-HMM	Autoregressive hidden Markov model
AT	Ataxia-telangectasia
BARS	Brief ataxia rating scale
CNN	Convolutional neural network
FRDA	Friedreich’s ataxia
HDP-HMM	Hierarchical Dirichlet process hidden Markov model
ICARS	International cooperative ataxia rating scale
ILOCA	Idiopathic late-onset cerebellar ataxia
IMU	Inertial measurement unit
MSA	Multiple system atrophy
MVN	Multivariate normal distribution
SARA	Scale for the assessment and rating of ataxia
SCA	Spinocerebellar ataxia
